# Intermittent hypoxia drives lung microbiome-metabolome remodeling to create a pro-inflammatory landscape in murine OSAHS

**DOI:** 10.3389/fmicb.2026.1797420

**Published:** 2026-06-24

**Authors:** Can-tang Zhang, Yu-xin Ye, Xin-xing Huang, Xiang-ju Wei, Lei Ji, Wen-hui Zhang, Jin Gao, Rui Chen

**Affiliations:** 1 Department of Respiratory and Critical Care Medicine, The Second Affiliated Hospital of Soochow University, Suzhou, Jiangsu, China; 2 Department of Respiratory and Critical Care Medicine, The Affiliated Hospital of Xuzhou Medical University, Xuzhou, Jiangsu, China; 3 Department of Neurobiology and Cellular Biology, Xuzhou Medical University, Xuzhou, Jiangsu, China

**Keywords:** bronchoalveolar lavage fluid (BALF), intermittent hypoxia (IH), lung microbiome, metabolome, obstructive sleep apnea-hypopnea syndrome (OSAHS)

## Abstract

**Background:**

Obstructive sleep apnea-hypopnea syndrome (OSAHS), characterized by intermittent hypoxia (IH), is associated with pulmonary complications. The specific mechanisms by which IH impacts the lung’s native microbiome and its functional metabolic output, however, remains largely uncharted.

**Methods:**

We established an OSAHS model in C57BL/6J mice using 4 weeks of IH exposure. Lung histology and inflammatory cytokines in bronchoalveolar lavage fluid (BALF) were assessed. We performed an integrated analysis of the lung microenvironment using 16S rRNA sequencing for the microbiota and LC-MS for the metabolome.

**Results:**

IH induced significant lung inflammation, evidenced by inflammatory infiltration and a polarized cytokine profile (elevated IL-1β, IL-6, TNF-α; decreased IL-10). Microbiome analysis revealed IH-driven dysbiosis, characterized by a marked shift in community structure and enrichment of pro-inflammatory taxa (e.g., Bacillota, *Mycoplasma*). Concurrently, metabolomic profiling uncovered widespread disturbances, with significant alterations in 500 metabolites. Key changes included rises in pro-inflammatory molecules (e.g., stachydrine) and falls in protective mediators (e.g., prostaglandin E2, embelin). Pathway analysis indicated these metabolites were enriched in niacin metabolism, inflammatory mediator regulation of TRP channels, and neuroactive ligand-receptor interactions. Crucially, correlation analysis delineated a robust interplay between the specific IH-altered microbial taxa and the disturbed metabolic pathways, suggesting a coordinated microenvironmental response.

**Conclusion:**

Our integrated analysis reveals a compelling association between the lung microbiota and metabolome, suggesting their potential role as a cooperative factor associated with pulmonary inflammation in OSAHS. This study establishes a valuable resource and outlines a framework for future mechanistic and therapeutic exploration.

## Graphical Abstract

**Figure:**
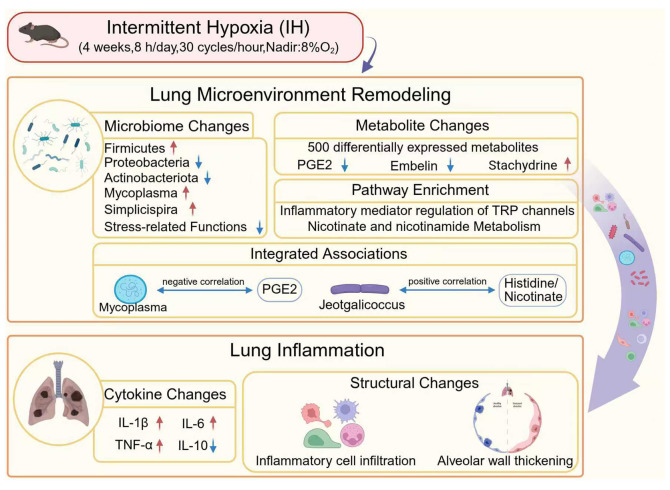
Intermittent hypoxia (IH) drives remodeling of the lung microbiome (e.g., increased Mycoplasma and Firmicutes) and metabolome (e.g., decreased PGE2, altered TRP/nicotinate pathways). A negative correlation between Mycoplasma and PGE2 links these changes, contributing to a pro-inflammatory landscape with elevated IL-1β/IL-6/TNF-α, reduced IL-10, and structural lung damage in murine OSAHS.

## 1 Introduction

Obstructive sleep apnea hypopnea syndrome (OSAHS) is a prevalent sleep-related breathing disorder characterized by recurrent upper airway collapse, leading to intermittent hypoxia (IH). Affecting nearly a billion adults globally, OSAHS poses a significant health burden and is a well-established driver of systemic multi-organ damage through IH-induced oxidative stress and inflammation (Benjafield et al., 2019; Dong et al., 2023; Lyons et al., 2020; Tondo et al., 2023). Among its complications, OSAHS-associated lung pathology represents a clinically significant yet mechanistically underexplored facet of the disease.

Beyond the systemic effects, there is growing appreciation for the role of local tissue microenvironments in health and disease. The lung, once considered sterile, are now known to host a diverse microbial community known as the lung microbiome. In healthy individuals, the dominant phyla include Bacillota, Bacteroidota, Pseudomonadota, Actinobacteriota, and Fusobacteriota (Belizário et al., 2023). In chronic respiratory diseases such as asthma and idiopathic pulmonary fibrosis, this community undergoes dysbiosis, often characterized by reduced microbial diversity and an enrichment of Pseudomonadota (Zhou et al., 2024). The murine lung microbiome shares broadly similar dominant phyla with the human lung, though species-level composition and microbial density differ (Hoytema van Konijnenburg et al., 2024). The lung microbiome plays a crucial role in maintaining immune homeostasis and barrier function (Dickson et al., 2016; Yang et al., 2020; Zhao et al., 2023). The stability of this ecosystem is vital; its disruption, termed dysbiosis, has been implicated in the pathogenesis of various respiratory diseases, such as idiopathic pulmonary fibrosis and asthma (Amati et al., 2022; Budden et al., 2019; Wang et al., 2016; Yi et al., 2022).

A key trigger of such dysbiosis is environmental stress. In OSAHS, IH is a potent, recurrent physiological stressor. While studies have shown that IH can induce intestinal dysbiosis and compromise gut barrier integrity—a phenomenon linked to remote organ injury via the gut-lung axis (Wu et al., 2022; Barcik et al., 2020)—a critical gap remains. The direct impact of IH on the local lung microbiome itself, and the functional consequences thereof, has been largely overlooked. Given that the lung microbiome interacts with the host primarily through bacterially derived metabolites (Segal et al., 2016; Whiteson et al., 2014), any IH-induced microbial shifts would likely manifest in altered metabolic output, potentially directly fueling local inflammation. Beyond its effects on the microbiome, IH has also been shown to trigger oxidative stress and cell death pathways (e.g., apoptosis, pyroptosis), leading to the release of damage-associated molecular patterns that can amplify inflammatory responses (Lv et al., 2024; Ou et al., 2025).

We therefore hypothesized that chronic IH remodels the lung microbiome and its associated metabolome, thereby contributing to the pro-inflammatory state observed in OSAHS-related lung injury. To test this, we employed an integrated multi-omics approach combining 16S rRNA gene sequencing and LC-MS metabolomics on bronchoalveolar lavage fluid from a murine IH model.

This study is the first to comprehensively profile and correlate simultaneous alterations in the lung microbiota and metabolome under IH, suggesting a potential microbiota–metabolite–inflammation axis in OSAHS-related lung pathology. This work provides a foundational resource for understanding local pulmonary pathophysiology in OSAHS and highlights potential therapeutic targets for mitigating lung damage.

## 2 Materials and methods

### 2.1 Animals and ethical statement

Sixteen male C57BL/6J mice (8 weeks old, 20–25 g) were obtained from the Experimental Animal Center of Xuzhou Medical University. Mice were individually housed under a 12-h light/dark cycle (lights on from 6:00 to 18:00) in a controlled environment (temperature: 25 °C ± 1 °C; humidity: 55% ± 5%) with ad libitum access to food and water. After 1 week of acclimatization, all experimental procedures were conducted in strict accordance with the guidelines and were approved by the Animal Research Ethics Committee of Xuzhou Medical University.

### 2.2 Intermittent hypoxia (IH) modeling

Mice were randomly assigned to two groups (*n* = 8 per group): a normoxia (NO) control group and an IH group. The IH group was subjected to a cyclic hypoxia paradigm in a specialized chamber (Yuyan Science, China, S1008). According to previous studies (Chivers et al., 2024; Zhang et al., 2018), each cycle consisted of 60 seconds of normoxia (21% ± 0.5% O_2_), followed by a 20-s descent to a nadir of 7.5% ± 0.5% O_2_, which was maintained for 30 s, before returning to normoxia within 10 s. This 2-min cycle was repeated for 8 h per day (during the light phase) over 4 consecutive weeks. The severity of hypoxia (8% O2 nadir) and cycle frequency (30 cycles/hour) were chosen to mimic moderate-to-severe OSAHS in humans, based on previously published protocols. The NO group was housed in an identical chamber under continuous NO conditions for the same duration.

### 2.3 Sample collection

After the 4-week exposure, mice were deeply anesthetized with 1.25%Tribromoethanol at a dose of 0.2 mL/10 g within 2 h after the last IH cycle. Subsequent procedures, including dissection and tissue collection, were performed under aseptic conditions in a laminar flow biosafety cabinet. Bronchoalveolar lavage fluid (BALF) was collected by cannulating the trachea and instilling the lungs with sterile phosphate-buffered saline (PBS), which was pre-warmed, in two aliquots of 0.5 mL each. The collected BALF was then stored in sterile vials. The recovered BALF was centrifuged (4 °C, 500 × *g*, 10 min) to remove cells, and the supernatant was aliquoted and stored at −80 °C for subsequent cytokine, microbiome, and metabolomic analyses (Zhang et al., 2025a). While still under deep anesthesia, all mice were then euthanized by cervical dislocation as a secondary confirmatory method, in accordance with the AVMA Guidelines for the Euthanasia of Animals. The lungs were then harvested. The left lobe was fixed in 4% paraformaldehyde for histology, while the remaining tissues were snap-frozen in liquid nitrogen and stored at −80 °C.

### 2.4 Hematoxylin and eosin (H&E) staining and morphological analyses

Lung from the NO and IH mice were harvested, fixed in 4% paraformaldehyde for 24 h, and then dehydrated in a graded ethanol series (70%, 80%, 95%, 100%; 30 min each), cleared in xylene, embedded in paraffin using a Leica EG1150H paraffin embedding station (Leica Biosystems, Germany), sectioned at 5 μm thickness using a Leica RM2255 rotary microtome (Leica Biosystems, Germany), and mounted on glass slides. The sections were then dried at 37 °C overnight before staining. Paraffin tissue sections were deparaffinized in xylene and rehydrated through a graded ethanol series. Sections were then stained with hematoxylin (Beyotime, China, Catalog #C0105S) for 5 min, rinsed in tap water, differentiated in 0.3% acid alcohol for 10 s, and blued in Scott’s tap water substitute for 1 min. After rinsing, sections were counterstained with eosin for 2 min, dehydrated, cleared in xylene, and mounted with neutral resin. All steps were performed at room temperature according to the manufacturer’s protocol. The stained sections were examined using an Olympus microscope (model BX41 with a DP73 camera). For quantitative analysis of alveolar septal thickness, at least three non-consecutive lung sections per mouse were examined (Golden et al., 2021). Three random fields of view per section were captured at ×400 magnification. Alveolar septal thickness was manually measured using ImageJ software (NIH, United States), with at least five measurements per field of view. The mean value was calculated for each mouse and used for group comparison (*n* = 4 per group). Unpaired Student’s *t*-test was used to compare the NO and IH groups. Inflammatory cell infiltration was qualitatively assessed.

### 2.5 Cytokine measurement

The concentrations of interleukins (IL)-1β, IL-6, IL-10, and tumor necrosis factor-alpha (TNF-α) in cell-free BALF were quantified using commercial enzyme-linked immunosorbent assay (ELISA) kits (Proteintech Group, Inc., Wuhan, China; Catalog #: KE10003, KE10007, KE10103, KE10002, respectively) according to the manufacturer’s instructions. The optical density was measured using a microplate reader. BALF samples were used undiluted.

### 2.6 16 S rRNA gene sequencing and microbiome analysis

DNA extraction and sequencing: Microbial genomic DNA was extracted from BALF samples using the cetyltrimethylammonium bromide (CTAB) method (Wilson, 2001). The hypervariable V3–V4 region of the bacterial 16S rRNA gene was amplified with primers 341F (5′-CCTACGGGNGGCWGCAG-3′) and 805R (5′-GACTACHVGGGT ATCTAATCC-3′) (Herlemann et al., 2011). This primer pair targets the V3–V4 region and generates an amplicon of approximately 464 bp (excluding primers), which provides sufficient phylogenetic resolution for genus-level classification. The V3–V4 region was chosen because it is widely used for microbiome profiling and has comprehensive reference databases available (Klindworth et al., 2013). PCR products were purified, quantified, and paired-end sequenced (2 × 250 bp) on an Illumina NovaSeq platform according to standard protocols.

Bioinformatic Processing: Raw sequencing data were processed using QIIME2 (version 2020.6) (Bolyen et al., 2019). Briefly, primers and low-quality sequences were trimmed. Paired-end reads were merged, quality-filtered, and denoised using DADA2 to obtain amplicon sequence variants (ASVs) (Callahan et al., 2016), with the following parameters: truncLen = 240 bp for forward reads and 200 bp for reverse reads, maxEE = 2, truncQ = 2. Taxonomic assignment was performed against the SILVA database (version 138) (Quast et al., 2013). Alpha diversity (Shannon, Simpson, and Chao1 indices) was calculated using the diversity plugin within QIIME2. Beta diversity was assessed based on Jaccard and unweighted UniFrac distances using the beta-diversity plugin in QIIME2. To test for significant differences in beta diversity between groups, PERMANOVA (permutational multivariate analysis of variance) with 999 permutations was performed using the beta-group-significance plugin in QIIME2. Linear discriminant analysis Effect Size (LEfSe) was employed to identify differentially abundant taxa between groups using the LEfSe online tool^1^, with a logarithmic LDA score threshold of >2.0 for general comparisons and >3.5 for the final figure, and an alpha value of 0.05 for the factorial Kruskal-Wallis test (Fan et al., 2021).

The raw sequencing data have been deposited under BioProject accession number PRJCA030083 and OMIX accession number OMIX007874.

Functional and phenotypic prediction: Functional profiling of the lung microbiome was inferred using Reconstruction of Unobserved States (PICRUSt2, version 2.5.0^2^) based on the 16S rRNA gene amplicon sequence variants (Douglas et al., 2020). The pipeline integrates phylogenetic placement of ASVs, hidden-state prediction of gene family copy numbers, and metagenome functional inference against the integrated KEGG and COG databases. The mean nearest sequenced taxon index (NSTI) across all ASVs was 0.171 ± 0.746, indicating adequate phylogenetic resolution for functional prediction. The predicted COG categories were subsequently compared between groups using STAMP (version 2.1.3) with Welch’s *t*-test. Additionally, microbial community phenotypes were predicted using BugBase (Ward et al., 2025).

### 2.7 Metabolomic analysis

Metabolite extraction: A 200 μL aliquot of BALF was lyophilized. As BALF represents the alveolar lining fluid, the detected metabolome primarily reflects extracellular metabolites secreted or released into the airway lumen. Metabolites were then extracted using 400 μL of pre-chilled to 4 °C extraction solution (methanol:acetonitrile:water = 2:2:1, v/v) containing internal standards (2-chloro-L-phenylalanine, 10 μg/mL). The mixture was vortexed vigorously, sonicated for 10 min at 4 °C, and incubated at −40 °C for 1 h to precipitate proteins. After centrifugation at 12,000 rpm (15,557 × *g*, Eppendorf 5804R, Germany), 15 min, 4 °C, the supernatant was collected for LC-MS analysis (Fu et al., 2024).

LC-MS analysis: Metabolic profiling was performed using a Vanquish UHPLC system coupled to an Orbitrap Exploris 120 mass spectrometer (Thermo Fisher Scientific). Separation was achieved on a Waters ACQUITY UPLC BEH Amide column (2.1 × 50 mm, 1.7 μm) with mobile phase A (25 mM ammonium acetate and 25 mM ammonia in water) and B (acetonitrile). The gradient elution program was as follows: 0–0.5 min, 95% B; 0.5–7.0 min, 95%–65% B; 7.0–8.0 min, 65%–40% B; 8.0–9.0 min, 40% B; 9.0–9.1 min, 40%–95% B; 9.1–12.0 min, 95% B for re-equilibration. The total run time was 10 min per sample. The flow rate was 0.5 mL/min, and the column temperature was 40 °C (Zhang et al., 2025b). The mass spectrometer was operated in both positive and negative ionization modes. The ESI source conditions were set as follows: sheath gas flow rate, 50 Arb; auxiliary gas flow rate, 15 Arb; capillary temperature, 320 °C; full MS resolution, 60,000; MS/MS resolution, 15,000; collision energy, NCE 20/30/40; spray voltage, 3.8 kV (positive) or −3.4 kV (negative).

Data processing and annotation: Raw data were converted to mzXML format and processed using an in-house program developed in R and based on XCMS, as previously described (Zhou et al., 2022). Metabolite annotation was performed using BiotreeDB (version 3.0) against the HMDB database. Significantly altered metabolites were screened based on a variable importance in projection (VIP) score > 1 from the OPLS-DA model and a *p*-value < 0.05 from Student’s *t*-test (Chen et al., 2021). Pathway enrichment analysis was performed using the Pathway Analysis module of MetaboAnalyst 5.0^3^, with the Mus musculus KEGG pathway library as reference (Pang et al., 2021).

### 2.8 Statistical analysis

Data are presented as mean ± standard error of the mean (SEM). Statistical analyses were performed using GraphPad Prism 8.0 and R software. Differences between two groups were assessed by the two-tailed unpaired Student’s *t*-test (for cytokine data) or the non-parametric Wilcoxon rank-sum test (for alpha and beta diversity indices). Correlations between microbiome and metabolome data were evaluated using Spearman’s rank correlation coefficient. To minimize spurious associations from sparse or low-abundance taxa, only genera with a detection frequency of ≥50% in at least one group (NO or IH) were included in the Spearman correlation analysis. A *p*-value < 0.05 was considered statistically significant.

## 3 Results

### 3.1 IH induces structural damage and inflammatory infiltration in the lung

**FIGURE 1:**
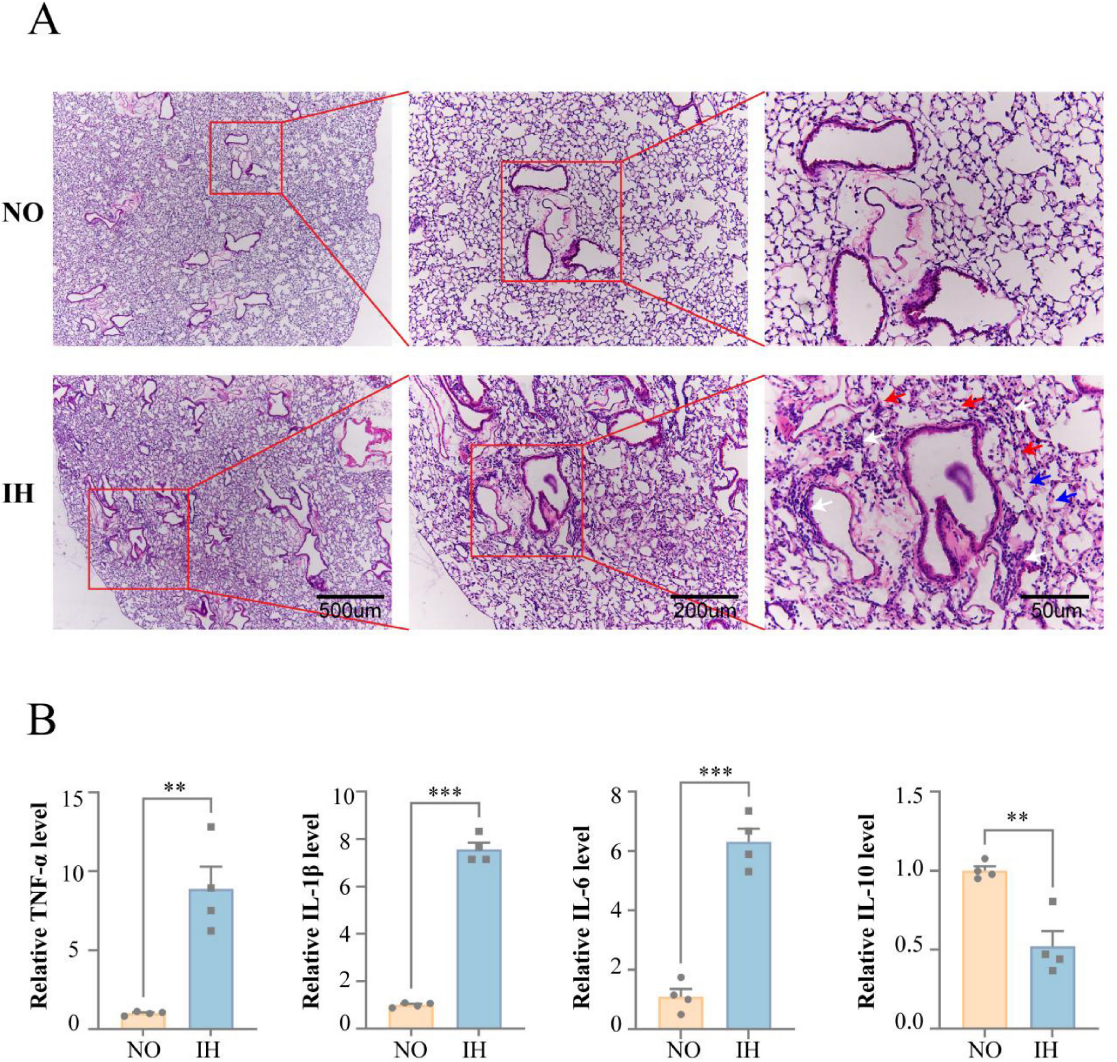
Intermittent hypoxia (IH) induces pulmonary inflammation and a pro-inflammatory cytokine profile. **(A)** Representative hematoxylin and eosin (H&E)-stained lung sections from mice exposed to normoxia (NO) or IH for 4 weeks. The IH group shows inflammatory cell infiltration (white arrows), thickened alveolar septa (red arrowheads), and slight bleeding (blue arrowheads). Scale bars, 500, 200, and 50 μm (from left to right). **(B)** Relative levels of the pro-inflammatory cytokines TNF-α, IL-1β, IL-6, and the anti-inflammatory cytokine IL-10 in bronchoalveolar lavage fluid (BALF), measured by ELISA and normalized to the NO group (set to 1). Statistical significance was determined by two-tailed unpaired Student’s *t*-test. Individual data points (*n* = 4 per group) are overlaid on the bar graphs to show the distribution of the data (**p < 0.01, ***p < 0.001).

To determine the structural impact of IH, we first examined lung histology. Qualitative histological assessment revealed that while lung architecture remained largely intact in NO controls, IH exposure induced apparent pathological changes, consistent with previous reports (Lee et al., 2017). These changes were characterized by noticeable inflammatory cell infiltration around the terminal bronchi, fragmentation of alveolar epithelial structures, and thickening of the alveolar walls (Figure 1A). Quantitative morphometric analysis further confirmed that IH exposure significantly increased alveolar septal thickness compared to the NO group (Supplementary Figure 1), in agreement with prior studies (Chou et al., 2019). The observed pattern of inflammatory cell infiltration and alveolar remodeling is characteristic of low-grade pulmonary inflammation. In line with this morphological evidence, analysis of BALF showed that IH triggered a marked pro-inflammatory shift in the pulmonary cytokine milieu. The levels of pro-inflammatory cytokines TNF-α, IL-1β, and IL-6 were significantly elevated, while the concentration of the anti-inflammatory cytokine IL-10 was markedly suppressed in the IH group compared to NO controls (Figure 1B). Together, these data confirmed that our IH model successfully recapitulated the well-documented pulmonary inflammatory state associated with OSAHS, thereby validating this model for the subsequent microbiome and metabolomic investigations.

### 3.2 IH remodels the diversity and composition of the lung microbiome

**FIGURE 2:**
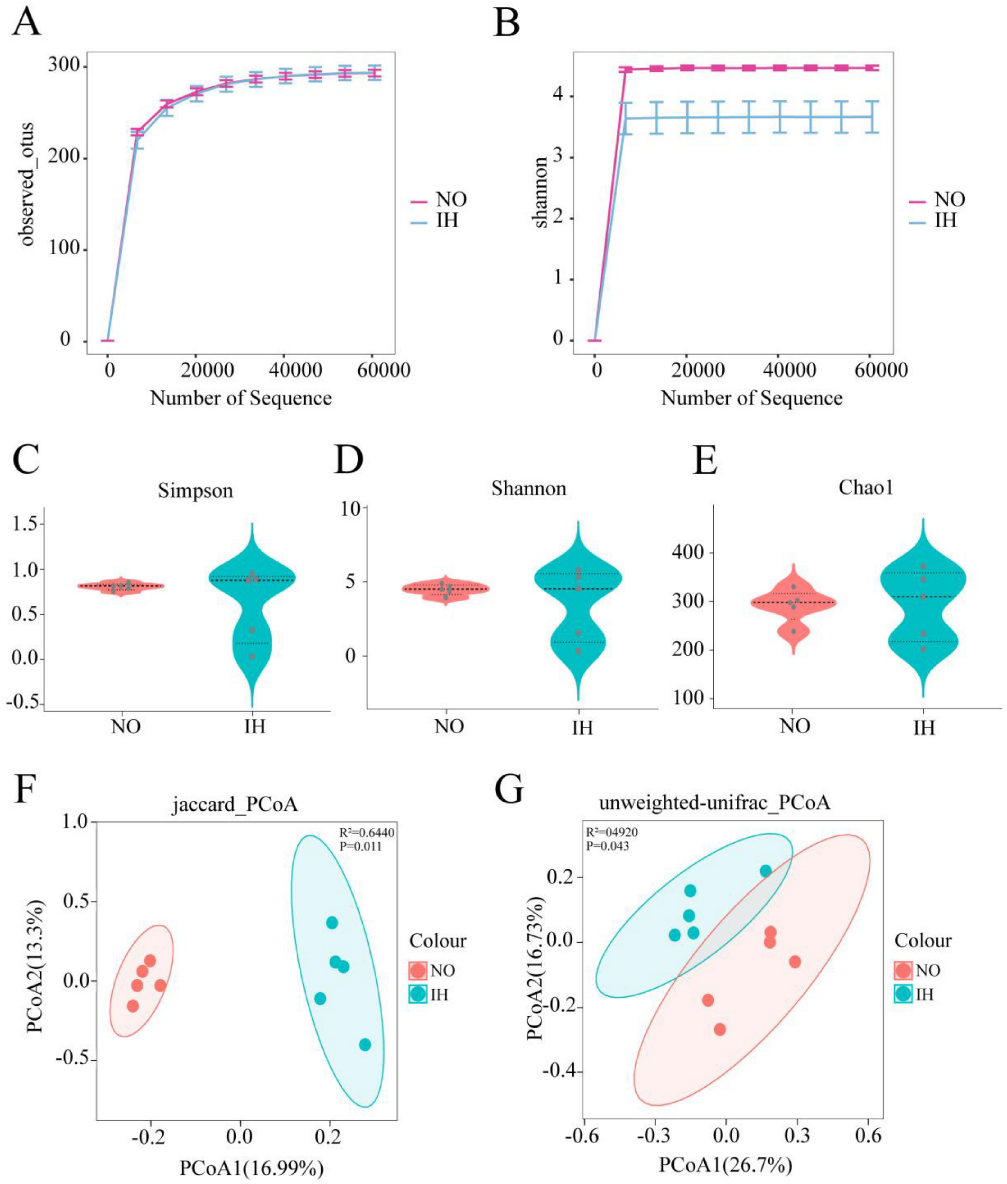
Intermittent hypoxia (IH) alters the diversity and structure of the lung microbial community. **(A,B)** Rarefaction curves of observed OTUs **(A)** and Shannon index **(B)** plateau, indicating adequate sequencing depth. **(C–E)** Alpha diversity of the lung microbiota assessed by **(C)** Simpson (NO: 0.81 ± 0.03, IH: 0.66 ± 0.33), **(D)** Shannon (NO: 4.47 ± 0.30, IH: 3.66 ± 2.03), **(E)** Chao1 (NO: 293.60 ± 28.15, IH: 294.37 ± 61.05), statistical significance was determined by the Wilcoxon rank-sum test, *p* > 0.05. **(F,G)** Beta diversity analyzed by principal coordinate analysis (PCoA) based on **(F)** Jaccard distance (R^2^ = 0.6440, *p* = 0.011) and **(G)** unweighted UniFrac distance (R^2^ = 0.4920, *p* = 0.043).

To assess the impact of IH on the pulmonary microbiota, we performed 16S rRNA gene sequencing analysis of BALF. The sequencing depth was evaluated by plotting rarefaction curves for Observed OTUs and the Shannon index. The curves for all samples reached a plateau (Figures 2A,B), indicating sufficient sequencing depth to capture the majority of microbial diversity present.

Based on this adequate sequencing depth, we compared the alpha diversity of lung microbiota between the IH and Control groups. Although the mean values of the Shannon and Simpson indices were lower in the IH group with greater intra-group variation, no statistically significant differences were observed between the two groups (Figures 2C,D). Similarly, the Chao1 index showed no significant difference between groups (Figure 2E), suggesting that intermittent hypoxia did not markedly alter the total species richness, and its effect may be more reflected in changes in species relative abundance rather than species gain or loss. Further analysis of community structure differences via beta diversity, using Principal Coordinate Analysis (PCoA) based on Jaccard and Unweighted Unifrac distances, revealed clear separation between the IH and Control groups (Figures 2F,G). PERMANOVA tests confirmed significant inter-group differences for the Jaccard distance (*p* = 0.011, R^2^ = 0.644), a finding supported by the Unweighted Unifrac distance results (*p* = 0.043, R^2^ = 0.492). These results indicate that intermittent hypoxia is a key driver altering the structure of the lung microbial community.

### 3.3 IH drives a pro-inflammatory shift in lung microbiome composition

**FIGURE 3:**
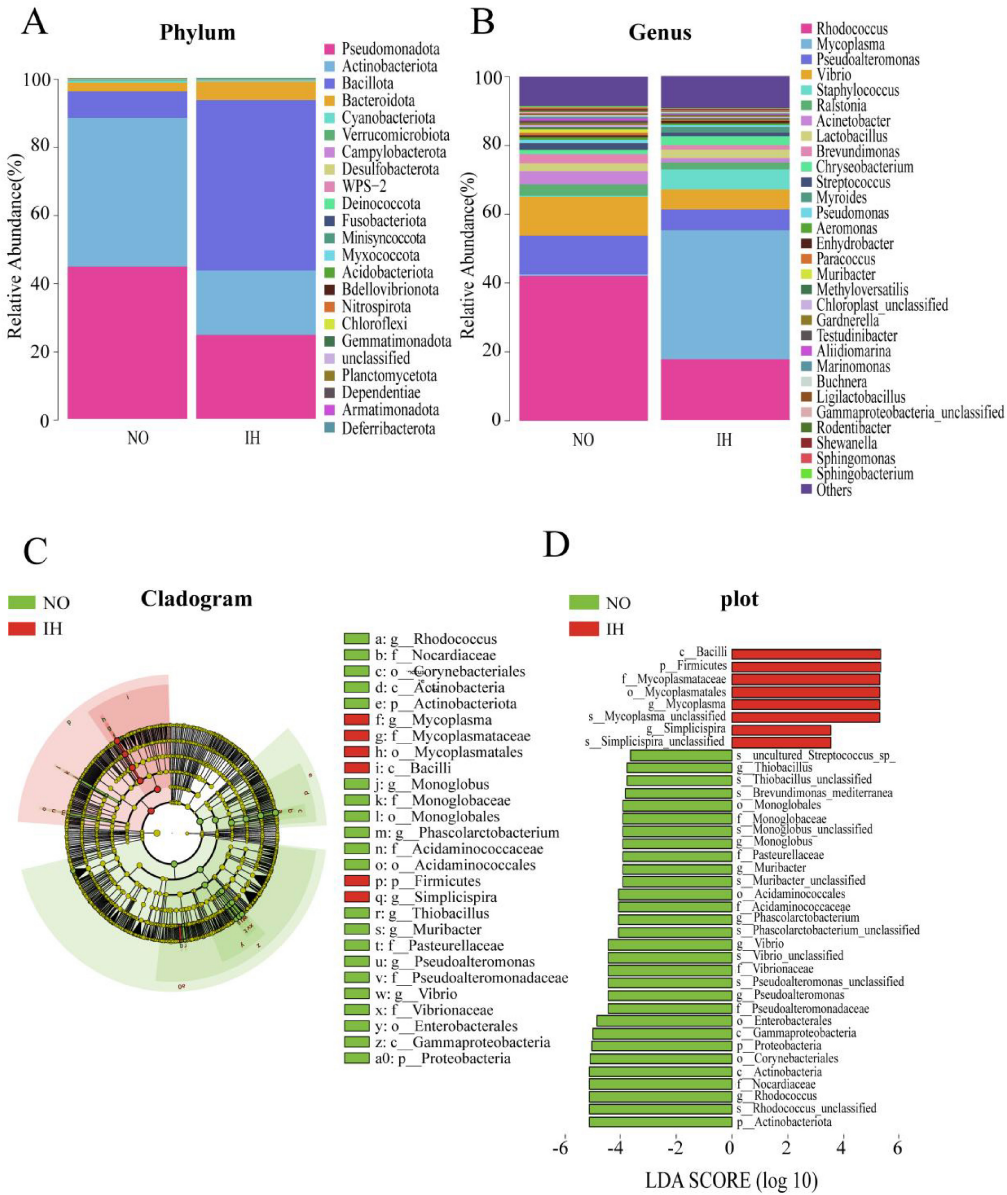
Intermittent hypoxia (IH) reshapes the taxonomic composition of the lung microbiota. **(A,B)** Relative abundance of bacterial **(A)** phyla and **(B)** genera in the lung microbiota of mice exposed to normoxia (NO) or IH. Only the top most abundant taxa are shown. **(C)** Cladogram generated from linear discriminant analysis effect size (LEfSe) analysis, showing the phylogenetic distribution of microbial taxa associated with the NO (green) and IH (red) groups. **(D)** Histogram of the LDA scores for bacterial taxa showing statistically significant differences between groups (LDA score > 3.5).

The restructuring of the lung microbiota was evident in specific taxonomic alterations. At the phylum level, the dominant communities were significantly reshaped by IH, with a marked decrease in the relative abundances of Pseudomonadota and Actinobacteriota, and a concurrent increase in Bacillota (Figure 3A). This expansion of Bacillota was primarily driven by the enrichment of genera such as *Mycoplasma* and *Staphylococcus* (Figure 3B). This shift resulted in a notable elevation of the Bacillota-to-Bacteroidota ratio, a microbial signature often associated with inflammatory states.

At the genus level, Linear discriminant analysis Effect Size (LEfSe) identified specific genera that were significantly enriched in the IH group, most notably *Mycoplasma* and *Simplicispira* (Figures 3C,D). Consistently, *Mycoplasma* is classified under the phylum Bacillota in the SILVA 138 database used in this study, and its significant expansion in the IH group was confirmed by genus-level profiling (Figure 3B). *Mycoplasma* has been independently linked to the pathogenesis of severe inflammatory lung diseases (Wang et al., 2024; Xue et al., 2023), supporting its potential relevance to the observed pulmonary inflammation. In addition, LEfSe also identified *Simplicispira*, a genus belonging to the phylum Pseudomonadota, as significantly enriched in the IH group, despite the overall decline of this phylum. Given its very low relative abundance (≤0.01% across samples), this statistical enrichment reflects more consistent detection of *Simplicispira* in the IH group and has negligible impact on the total Pseudomonadota abundance, which was primarily driven by reductions in other, more dominant members of this phylum.

### 3.4 IH reprograms the functional potential of the lung microbiome toward a stress-associated and virulent state

**FIGURE 4:**
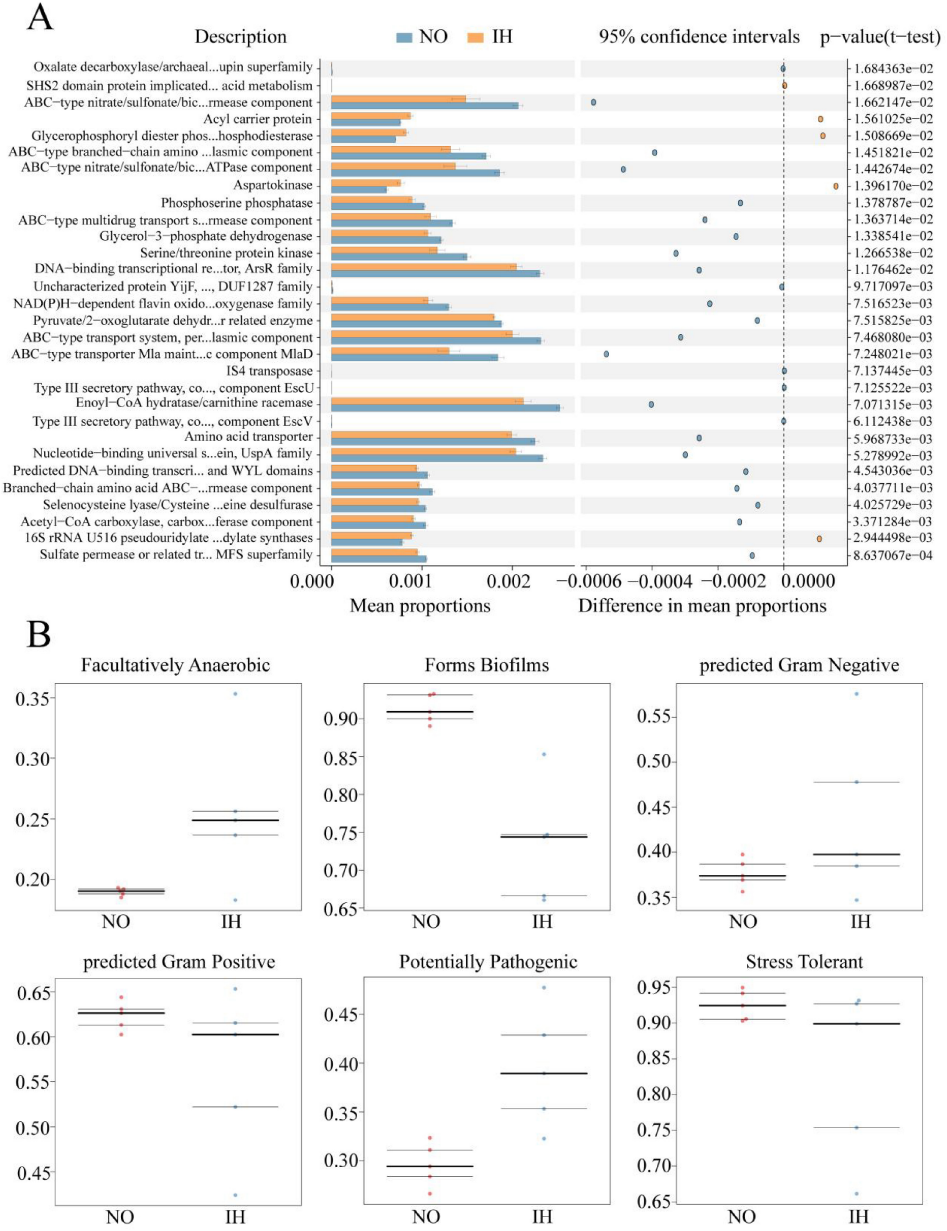
Predictive functional and phenotypic profiling of the lung microbiota under intermittent hypoxia (IH). **(A)** Differences in predicted metagenomic functions (Clusters of Orthologous Groups, COGs) between the normoxia (NO) and IH groups, analyzed by STAMP. The plot shows the top 30 COG categories with significant differences (Welch’s *t*-test, *p < 0.05; 95% confidence intervals shown). **(B)** Predicted relative abundances of microbial phenotypes based on BugBase analysis. Statistical significance was determined by the Wilcoxon rank-sum test. Gram-positive and Gram-negative categories in BugBase are predicted based on taxonomic annotations of amplicon sequence variants (ASVs); Gram-positive includes Bacillota and Actinobacteriota, while Gram-negative includes Pseudomonadota and Bacteroidota, among others.

To decipher the functional consequences of the observed microbial restructuring, we predicted the metagenomic capacities of the lung microbiota. PICRUSt2 analysis revealed that IH exposure was associated with a decreased relative abundance of key functional gene categories related to bacterial homeostasis and environmental sensing, including branched-chain amino acids transporters, universal stress proteins (UspA family), and ABC-type xylose transport systems (Figure 4A). These depleted functions are typically associated with Pseudomonadota and Actinobacteriota, the phyla that decreased under IH. Conversely, IH-enriched functions included acyl carrier protein and glycerophosphoryl diester phosphodiesterase (Figure 4A).

We next characterized the resulting phenotypic shifts using BugBase. This analysis revealed that the IH-remodeled microbiota was enriched for facultative anaerobes and predicted Gram-negative bacteria, while exhibiting a reduction in predicted Gram-positive bacteria, biofilm-forming, and stress-tolerant populations (Figure 4B). This phenotypic profile is consistent with a community adapted to a dysbiotic and potentially more inflammatory environment, as the increase in facultative anaerobes may reflect local hypoxia, and the shift in Gram-status can directly influence the host’s immune recognition. Collectively, these functional predictions illustrate that IH induces more than just taxonomic changes; it drives the lung microbiome toward a functionally distinct state that may actively contribute to disease pathophysiology.

### 3.5 IH induces profound remodeling of the lung metabolic landscape

**FIGURE 5:**
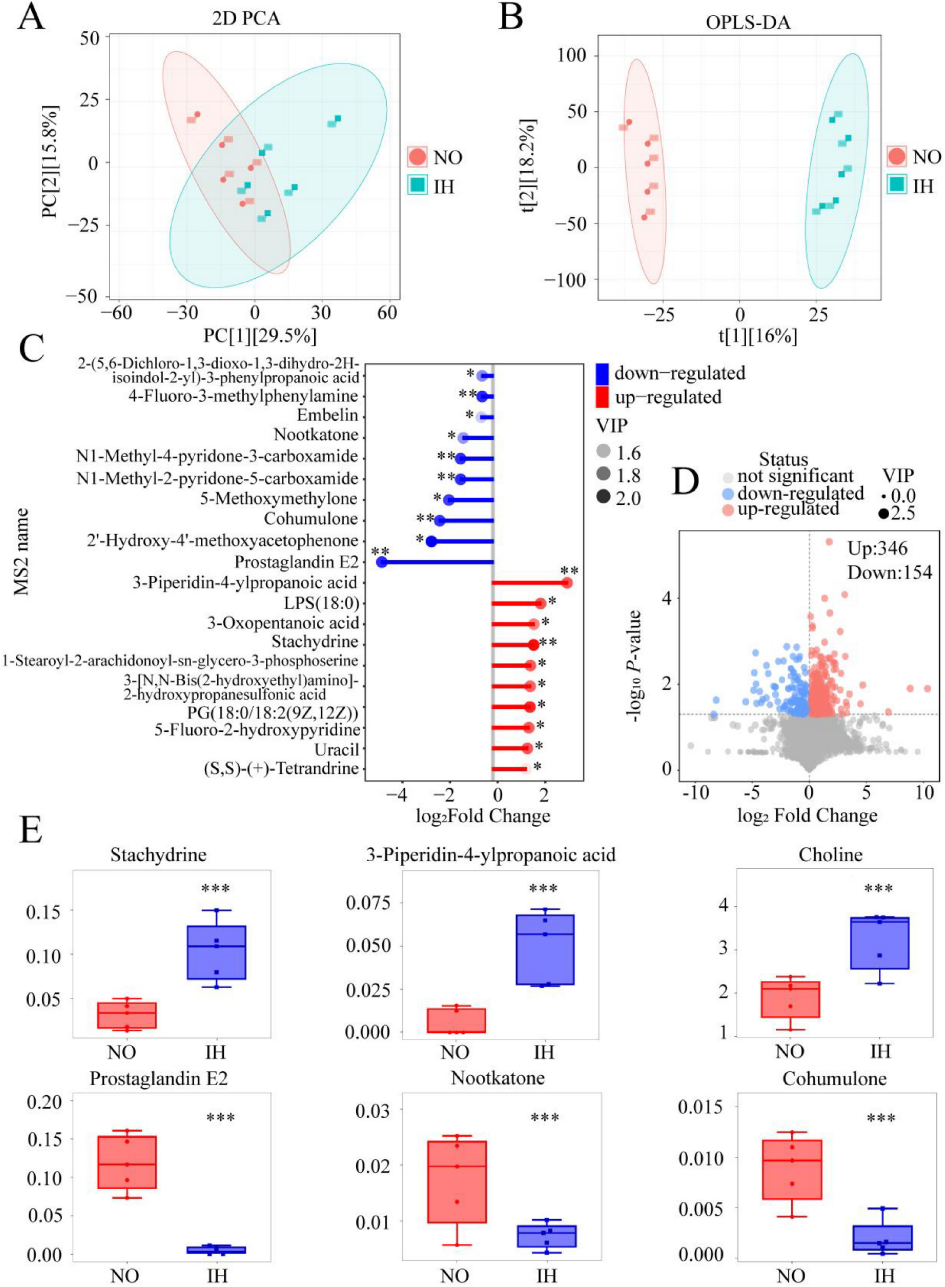
Intermittent hypoxia (IH) induces a profound shift in the lung metabolome. **(A)** Principal component analysis (PCA) score plot of the metabolomic profiles from bronchoalveolar lavage fluid (BALF). **(B)** Orthogonal partial least squares-discriminant analysis (OPLS-DA) score plot demonstrating separation between the normoxia (NO) and IH groups. The X-axis represents the predictive component t[1] (explaining 16% of the variance), and the Y-axis represents the first orthogonal component t[2] (explaining 18.2% of the variance). The OPLS-DA model generated one predictive and one orthogonal component, with the latter denoted as t[2] by the software. **(C)** Stack plot illustrating the overall abundance of differentially expressed metabolites. **(D)** Volcano plot of all detected metabolites. The X-axis shows the log_2_(fold change), and the Y-axis shows the –log_10_(*p*-value) from the Student’s *t*-test. Metabolites with significant changes (*p* < 0.05 and VIP > 1) are highlighted in red (up-regulated) and blue (down-regulated). **(E)** Box plots of the relative abundances of selected significantly altered metabolites. Statistical significance was determined by the Student’s *t*-test. (*p < 0.05, **p < 0.01, ***p < 0.001).

We next leveraged LC-MS-based metabolomics to determine how the IH-shaped microbial community and host cells collectively influence the lung’s biochemical environment. Principal component analysis (PCA) of the metabolomics data revealed a clear separation between the NO and IH groups (Figure 5A), a finding corroborated by the orthogonal partial least squares-discriminant analysis (OPLS-DA) model (Figure 5B). Based on the criteria of VIP > 1 from OPLS-DA and *p* < 0.05 (Student’s *t*-test), we identified 500 differentially abundant metabolites, with 346 increased and 154 decreased in the IH group (Figures 5C,D). Among the most significantly altered metabolites, we observed a marked increase in stachydrine and a pronounced decrease in prostaglandin E2 (PGE2) (Figure 5E) and embelin (Supplementary Figure 2). This widespread metabolic disturbance underscores the profound impact of IH on the lung’s biochemical milieu, implicating both pro-inflammatory and tissue-repair pathways.

### 3.6 Disrupted metabolic pathways link microbiome changes to inflammatory signaling

**FIGURE 6:**
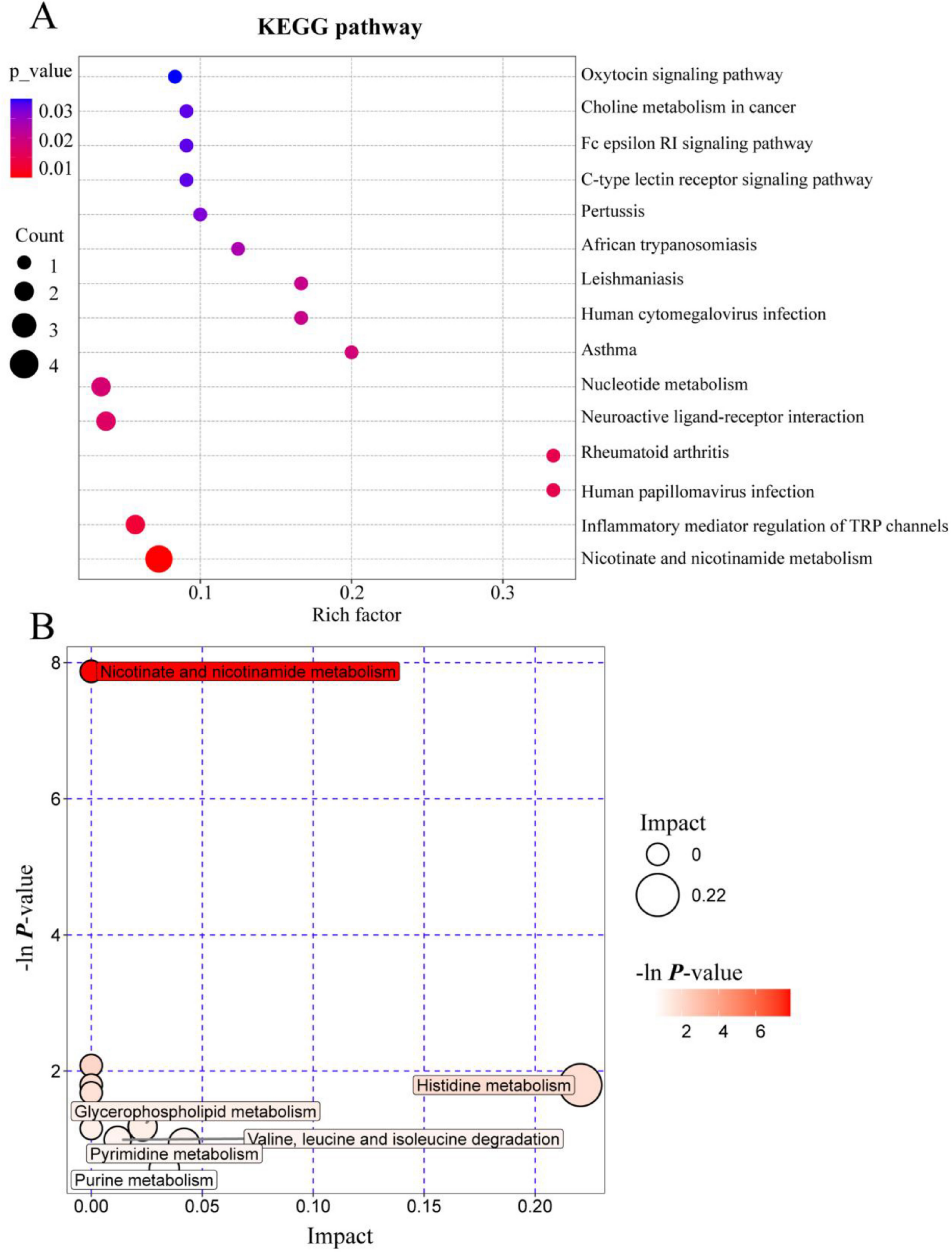
Pathway analysis of metabolomic alterations induced by intermittent hypoxia (IH). **(A)** KEGG pathway enrichment analysis (bubble plot). Bubble size represents the number of differential metabolites mapped to the pathway. Bubble color represents the *p*-value from enrichment analysis (darker red = more significant). Only pathways with *p* < 0.05 shown. **(B)** Alternative visualization of KEGG pathway analysis. Bubble size represents the pathway impact score from topology analysis (larger bubble = higher impact). Bubble color represents the –ln(*p*-value) (red = more significant).

To contextualize the metabolic alterations, we performed KEGG pathway enrichment analysis. This identified several key pathways significantly perturbed by IH, including Nicotinate and nicotinamide metabolism, inflammatory mediator regulation of TRP channels, and Neuroactive ligand-receptor interaction (Figure 6A). Pathway topology analysis further revealed perturbations in glycerophospholipid metabolism, purine/pyrimidine metabolism, and branched-chain amino acid degradation (Figure 6B). The enrichment of these specific pathways provides a crucial mechanistic link, suggesting that the IH-induced metabolic rewiring directly engages known inflammatory and cellular stress response signaling cascades within the lung.

### 3.7 An integrated correlation network implicates specific microbiome-metabolite interactions in inflammation

**FIGURE 7:**
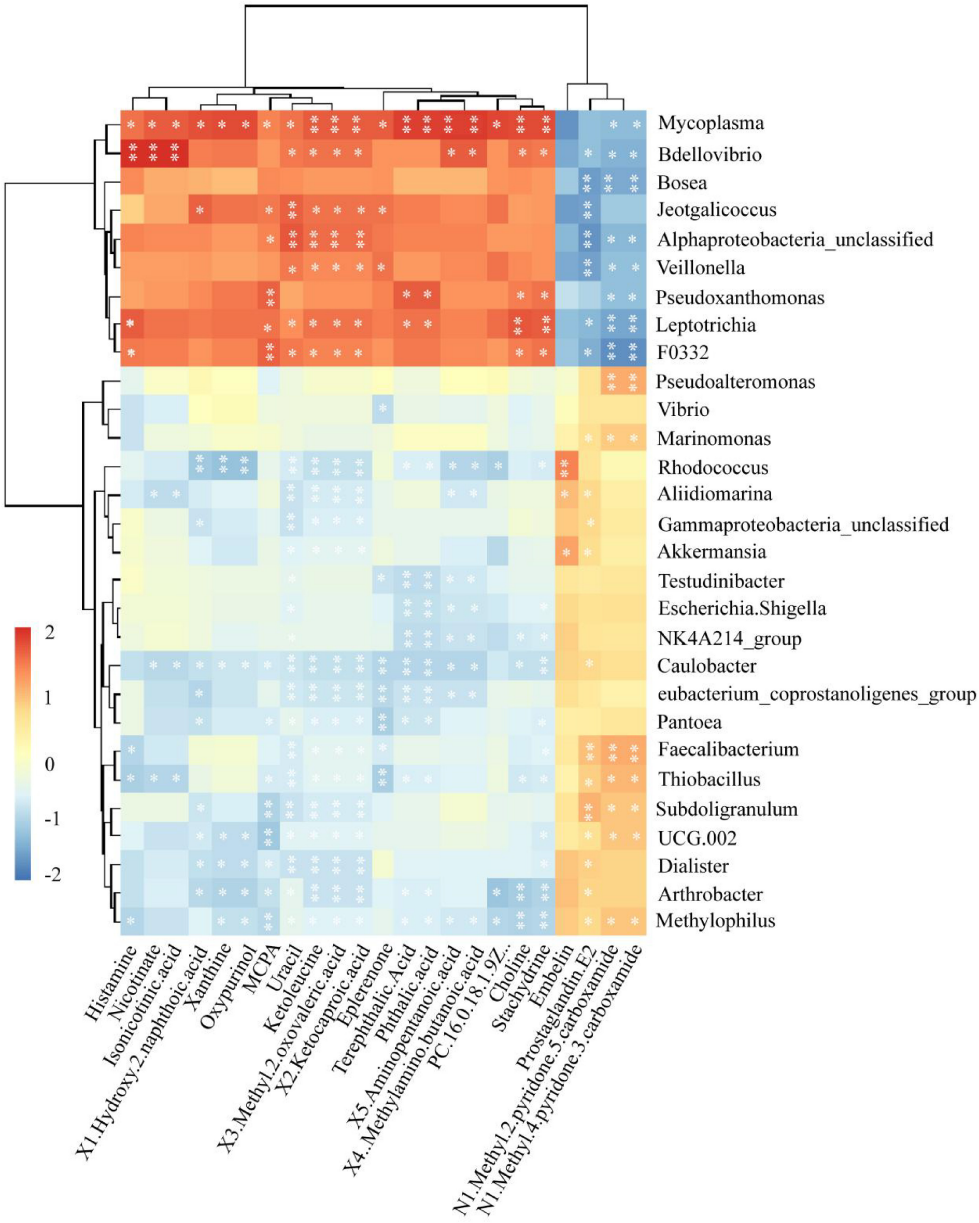
Integrated correlation network between the lung microbiome and metabolome. Heatmap showing Spearman’s correlation coefficients between the relative abundance of significantly altered microbial genera (rows) and the levels of significantly altered metabolites (columns) in bronchoalveolar lavage fluid (BALF). Only genera with a detection frequency of ≥50% in at least one group (NO or IH) were included in the analysis. Statistical significance is indicated as *p < 0.05, **p < 0.01. Red indicates positive correlations, and blue indicates negative correlations.

Finally, to directly explore the interplay between the remodeled lung microbiota and the disturbed metabolome, we conducted Spearman’s correlation analysis. This integrated approach revealed a robust network of associations between specific microbial genera and BALF metabolites (Figure 7). Strikingly, the anti-inflammatory metabolite PGE2, which was significantly depleted in the IH group, exhibited strong negative correlations with several genera enriched by IH, such as *Mycoplasma*. Conversely, the relative abundance of *Jeotgalicoccus* was strongly positively correlated with the levels of histidine and nicotinate. These data yield a set of high-priority, specific microbiome-metabolite interactions that form a plausible biochemical bridge linking IH-induced dysbiosis to the observed inflammatory phenotype.

## 4 Discussion

Our study provides the first integrated view of how IH remodels the lung microenvironment by simultaneously disrupting the microbiome and metabolome, thereby fostering a pro-inflammatory state. By moving beyond simple association studies, our multi-omics approach reveals coordinated alterations in the lung microbiota and metabolome that are associated with pulmonary inflammation, offering a novel framework for understanding OSAHS-associated lung pathology.

We first confirmed that IH establishes a low-grade inflammatory milieu in the lung, evidenced by histological damage and a polarized cytokine profile. This profile was characterized by a significant increase in pro-inflammatory mediators (IL-1β, IL-6, TNF-α)—with IL-1β being particularly notable for its ability to alter lung fibroblast function and promote chronic inflammation (Senoo et al., 2023)—and a concurrent decrease in the anti-inflammatory cytokine IL-10, a key regulator of inflammatory gene expression (Mishra et al., 2025). This finding sets the stage for investigating upstream drivers.

Intermittent hypoxia exposure was associated with marked dysbiosis, which may contribute to the inflammatory milieu. of the lung microbiota. The observed ecological shifts — increased diversity, enrichment of the pro-inflammatory phylum Bacillota (Li et al., 2019) and the genus *Mycoplasma* (a known instigator of severe lung inflammation (Wang et al., 2024; Xue et al., 2023), and a decline in Actinobacteriota (a phylum containing bacteria with immunoregulatory functions (Gavzy et al., 2023) — collectively point to a community structure predisposed to inflammation. This trend was further corroborated by a reduction in other taxa, including Pseudomonadota, which is often associated with airway inflammation (Guan et al., 2018), and the potentially pathogenic genus Pseudomonas (Gramegna et al., 2023), indicating a overall shift of the microbial community toward a pro-inflammatory phenotype. Our finding of decreased Pseudomonadota is consistent with IH rodent models (Lu et al., 2018), though clinical studies report Proteobacteria enrichment in OSAHS patients (Moreno-Indias et al., 2015). Several factors may explain this discrepancy: species differences in immune response and baseline microbiome composition between mice and humans; the simplified nature of the IH model (pure hypoxia) versus the complex pathophysiology of human OSAHS (which includes sleep fragmentation, intrathoracic pressure swings, and sympathetic activation); and differences in sampling sites (BALF in our study vs. other respiratory samples in clinical studies).

The significance of this shift is amplified by functional predictions. Crucially, this restructured microbiota exhibits a disturbed functional potential, with downregulation of genes for homeostasis (e.g., amino acid transporters, universal stress proteins) and upregulation of pathways like acyl carrier protein synthesis, potentially exacerbating oxidative stress (Chen et al., 2018; Luo et al., 2023; Sorbara and Pamer, 2019). Furthermore, the phenotypic prediction of an increase in facultative anaerobes and a shift in the predicted Gram-stain pattern likely reflects and adapts to local tissue hypoxia (Byndloss et al., 2017; Pickard et al., 2017). Thus, IH changes both microbial identity and functional capacity, creating a meta-inflammatory trigger.

The most direct mechanistic link between microbial dysbiosis and host inflammation is likely mediated by the metabolome. Our LC-MS analysis captured a profound metabolic rewiring. The significant downregulation of prostaglandin E2 (PGE2), a key lipid mediator whose synthesis is tightly regulated by ROS levels (Shim and Lim, 2025), reflects enhanced oxidative stress under IH conditions. Moreover, the marked elevation of choline in BALF raises the possibility that IH disrupts alveolar epithelial integrity, leading to respiratory barrier damage and leakage of cellular components, as choline is an essential component of cell membrane phospholipids (Kenny et al., 2025; van der Veen et al., 2017).

Our KEGG pathway analysis revealed that nicotinate and nicotinamide metabolism was the most significantly enriched pathway under IH. This pathway is central to NAD^+^ homeostasis, which is critical for ROS scavenging, DNA repair, and cell survival (Cantó et al., 2015). Notably, previous studies have demonstrated that chronic intermittent hypoxia disrupts NAD^+^ metabolism, leading to oxidative stress and cellular damage (Fan et al., 2023). NAD^+^ depletion has also been shown to trigger cell death, including apoptosis and pyroptosis (Sundaram et al., 2024). Disruption of this pathway by IH would therefore promote NAD^+^ depletion, exacerbate oxidative stress, and trigger cell death, thereby releasing cell-free DNA (cfDNA) into the extracellular space. Released cfDNA is a well-established agonist of the cGAS/STING pathway, which drives type I interferon-dependent inflammation (Deng et al., 2022). Thus, our metabolomic data provide a plausible link between IH-induced cell damage and the cGAS/STING-mediated inflammatory cascade.

The dual depletion of PGE2 and Embelin is particularly telling, as both are renowned for their anti-inflammatory and tissue-repair properties (Basha et al., 2022; Cheng et al., 2021; Yao et al., 2022). Their loss may compromise the lung’s intrinsic capacity to resolve inflammation. Among the elevated metabolites, we noted an increase in Stachydrine, potentially a compensatory anti-inflammatory response (He et al., 2024), and lipopolysaccharide (LPS), a potent driver of inflammation via NF-κB activation (Tang et al., 2021). This is complemented by the enrichment of metabolic pathways like “Inflammatory mediator regulation of TRP channels” (Zergane et al., 2021) and “Nicotinate and nicotinamide metabolism” (Lei et al., 2025), which directly connect these metabolite shifts to known pro-inflammatory signaling cascades and oxidative stress responses (Liu et al., 2025; Mansoori et al., 2025; Moro et al., 2020). Perturbations in glycerophospholipid metabolism, purine/pyrimidine metabolism, and branched-chain amino acid degradation further suggest that IH triggers a systemic metabolic stress response beyond the lung (Alotaibi et al., 2025; Zhou et al., 2019). These systemic metabolic changes raise the possibility that IH-induced cell damage may also occur in extra-pulmonary organs, such as the intestine, potentially contributing to the overall inflammatory state via the gut-lung axis. Other significantly altered metabolites, such as 3-piperidin-4-ylpropanoic acid, likely represent exogenous or microbial-derived compounds, as their structures are not typical of mammalian endogenous metabolism.

The pivotal question—how the microbial and metabolic changes are interconnected— is addressed by our correlation analysis. The strong negative correlation between PGE2 and pro-inflammatory genera like *Mycoplasma* suggests a potential link between *Mycoplasma* enrichment and reduced PGE2 levels, consistent with previous studies showing interactions between *Mycoplasma* and PGE2 in the lung (Goto et al., 2020; Wu et al., 2009). Conversely, the predator bacterium Bdellovibrio was positively correlated with numerous metabolites, and its expansion may influence microbial community structure and metabolite pools (Cavallo et al., 2021). These specific microbiome-metabolite interactions form a plausible biochemical bridge linking IH-induced dysbiosis to the inflammatory phenotype.

It should be noted that the current study cannot distinguish whether the observed microbial changes are a cause or a consequence of pulmonary inflammation. IH is known to directly induce host cellular stress and inflammatory responses, which could in turn shape the local microbial community. A bidirectional interaction between the lung microbiome and host inflammatory responses is likely to exist.

## 5 Limitations and future perspectives

This study has several limitations. First, our findings are primarily based on correlative analyses, and future experimental studies are needed for mechanistic validation. Our correlational design cannot establish whether dysbiosis precedes or follows inflammation; a bidirectional interaction is likely. We acknowledge that bioinformatic statistical controls cannot substitute for independent biological validation. Therefore, direct experimental interrogation of the key pathways implicated in this study is warranted in future investigations. As an exploratory work, the present findings provide a basis for such validation studies. In addition, we did not perform BALF total protein quantification or transmission electron microscopy to directly assess alveolar epithelial damage and repair, which would provide more direct evidence of respiratory barrier disruption.

Second, this model recapitulates the core feature of intermittent hypoxia and reproduces systemic inflammation and dysbiosis consistent with clinical observations (Liu et al., 2024; Tang et al., 2022). However, human OSAHS pathophysiology is more complex, encompassing sleep fragmentation, intrathoracic pressure swings, and sympathetic overactivation, and inherent species differences exist in immune systems, lung anatomy, and microbiome composition. Therefore, our findings are primarily confined to hypoxia-mediated effects. It should also be noted that there is currently no established cross-species scaling standard for IH parameters between mice and humans; functional modeling remains the mainstream paradigm in the field. Future clinical validation will be essential to assess translational relevance.

Third, while previous studies have shown that IH can induce gut barrier dysfunction and intestinal dysbiosis (Li et al., 2023; Xue et al., 2025), our study design (direct analysis of BALF) primarily captures local pulmonary changes. Future studies integrating fecal and BALF sampling with plasma metabolomics will be essential to systematically dissect the role of the gut-lung axis in OSAHS-associated lung injury.

Fourth, the baseline BALF samples were not collected prior to IH exposure. While all mice were randomly assigned from a genetically identical and uniformly housed cohort—the standard practice to ensure baseline comparability—we cannot formally rule out pre-existing differences in the lung microbiome. Future studies incorporating baseline sampling would further strengthen causal inferences.

Finally, validation in clinical OSAHS patient cohorts is essential. Nonetheless, our work definitively shifts the paradigm from viewing the lung as a passive victim of IH to recognizing it as a site of active, complex interplay between microbiota and host metabolism. This lung microbiota-metabolite-inflammation axis opens new therapeutic avenues, suggesting that interventions aimed at rebalancing the lung microenvironment could mitigate OSAHS-related lung injury.

## 6 Conclusion

In summary, our integrated multi-omics analysis unveils a previously unrecognized dimension of OSAHS pathophysiology: IH is associated with the lung microenvironment accompanied by co-dependent dysbiosis of the microbiota and a disruption of the metabolome. We demonstrate that these changes are not independent but are intricately linked, potentially contributing to a self-amplifying loop associated with disruption of immune homeostasis and associated with persistent pulmonary inflammation. This work suggests the lung microbiota-metabolite-inflammation axis as a factor involved in OSAHS-related lung damage, providing a novel conceptual framework and a solid foundation for future mechanistic and therapeutic exploration. Based on our pathway analyses, TRP channel modulators, nicotinate metabolism modulators, and microbiome-targeted interventions represent potential therapeutic strategies for mitigating OSAHS-associated lung inflammation.

## Data Availability

The datasets presented in this study can be found in online repositories. The names of the repository/repositories and accession number(s) can be found below: https://ngdc.cncb.ac.cn/omix/, OMIX007874 and https://www.ncbi.nlm.nih.gov/, PRJNA1151210.

## Footnotes

1
https://huttenhower.sph.harvard.edu/lefse/

2
https://github.com/picrust/picrust2

3
https://www.metaboanalyst.ca

## Supplementary material

Supplementary Figure 1 Quantitative analysis of alveolar septal thickness. Bar graph showing the alveolar septal thickness in the NO and IH groups. Data are presented as mean ± SEM. ****p* < 0.001, unpaired *t*-test.

Supplementary Figure 2 Relative abundance of embelin in bronchoalveolar lavage fluid (BALF) from mice exposed to normoxia (NO) or intermittent hypoxia (IH). Statistical significance was determined by the Student’s *t*-test (**p* < 0.05).
