# Supplementary figures and images for: Intermittent hypoxia drives lung microbiome-metabolome remodeling to create a pro-inflammatory landscape in murine OSAHS
Source: Front Microbiol. 2026 Jun 24;17:1797420. doi: 10.3389/fmicb.2026.1797420 (PMC13341692; doi:10.3389/fmicb.2026.1797420)

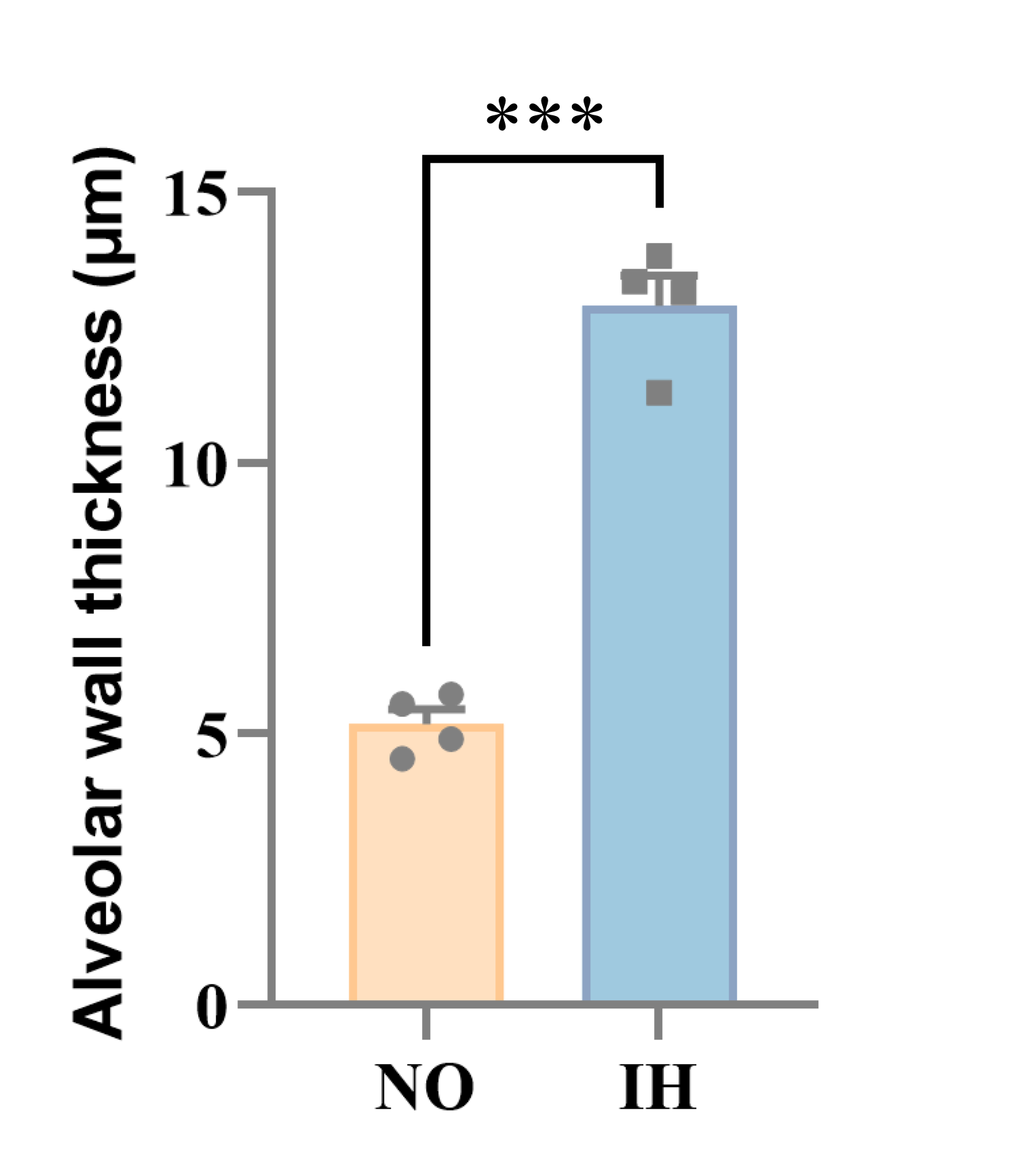

Supplement: Supplementary Figure 1 — Quantitative analysis of alveolar septal thickness. Bar graph showing the alveolar septal thickness in the NO and IH groups. Data are presented as mean ± SEM. ***p < 0.001, unpaired t-test. [file Image_1.tif]

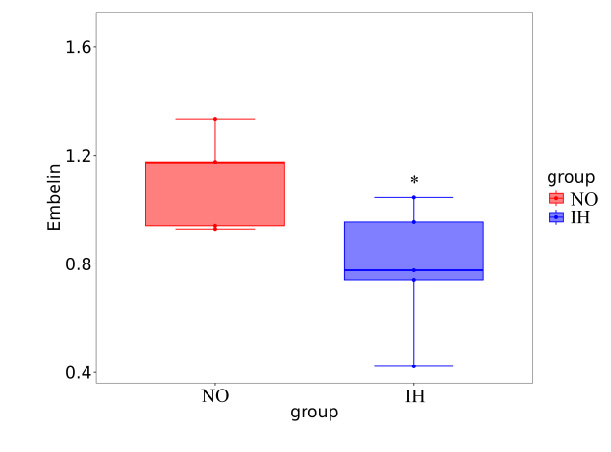

Supplement: Supplementary Figure 2 — Relative abundance of embelin in bronchoalveolar lavage fluid (BALF) from mice exposed to normoxia (NO) or intermittent hypoxia (IH). Statistical significance was determined by the Student’s t-test (*p < 0.05). [file Image_2.jpeg]
